# Modulation of the electronic structure and thermoelectric properties of orthorhombic and cubic SnSe by AgBiSe_2_ alloying†

**DOI:** 10.1039/d1sc03696c

**Published:** 2021-08-31

**Authors:** Sushmita Chandra, Raagya Arora, Umesh V. Waghmare, Kanishka Biswas

**Affiliations:** New Chemistry Unit, Jawaharlal Nehru Centre for Advanced Scientific Research (JNCASR) Jakkur P.O. Bangalore 560064 India kanishka@jncasr.ac.in; Chemistry and Physics of Materials Unit, Jawaharlal Nehru Centre for Advanced Scientific Research (JNCASR) Jakkur P.O. Bangalore 560064 India; Theoretical Sciences Unit, Jawaharlal Nehru Centre for Advanced Scientific Research (JNCASR) Jakkur P.O. Bangalore 560064 India; School of Advanced Materials and International Centre for Materials Science, Jawaharlal Nehru Centre for Advanced Scientific Research (JNCASR) Jakkur P.O. Bangalore 560064 India

## Abstract

Recently, single-crystals of tin selenide (SnSe) have drawn immense attention in the field of thermoelectrics due to their anisotropic layered crystal structure and ultra-low lattice thermal conductivity. Layered SnSe has an orthorhombic crystal structure (*Pnma*) at ambient conditions. However, the cubic rock-salt phase (*Fm*3̄*m*) of SnSe can only be stabilized at very high pressure and thus, the experimental realization of the cubic phase remains elusive. Herein, we have successfully stabilized the high-pressure cubic rock-salt phase of SnSe by alloying with AgBiSe_2_ (0.30 ≤ *x* ≤ 0.80) at ambient temperature and pressure. The orthorhombic polycrystalline phase is stable in (SnSe)_1−*x*_(AgBiSe_2_)_*x*_ in the composition range of 0.00 ≤ *x* < 0.28, which corresponds to narrow band gap semiconductors, whereas the band gap closes upon increasing the concentration of AgBiSe_2_ (0.30 ≤ *x* < 0.70) leading to the cubic rock-salt structure. We confirmed the stabilization of the cubic structure at *x* = 0.30 and associated changes in the electronic structure using first-principles theoretical calculations. The pristine cubic SnSe exhibited the topological crystalline insulator (TCI) quantum phase, but the cubic (SnSe)_1−*x*_(AgBiSe_2_)_*x*_ (*x* = 0.33) showed a semi-metallic electronic structure with overlapping conduction and valence bands. The cubic polycrystalline (SnSe)_1−*x*_(AgBiSe_2_)_*x*_ (*x* = 0.30) sample showed n-type conduction at room temperature, while the orthorhombic (SnSe)_1−*x*_(AgBiSe_2_)_*x*_ (0.00 ≤ *x* < 0.28) samples retained p-type character. Thus, by optimizing the electronic structure and the thermoelectric properties of polycrystalline SnSe, a high *zT* of 1.3 at 823 K has been achieved in (SnSe)_0.78_(AgBiSe_2_)_0.22_.

## Abstract 2

AgBiSe_2_ alloying in SnSe tailors its crystal and electronic structures, which boost its thermoelectric figure of merit to 1.3.

## Graphical abstract

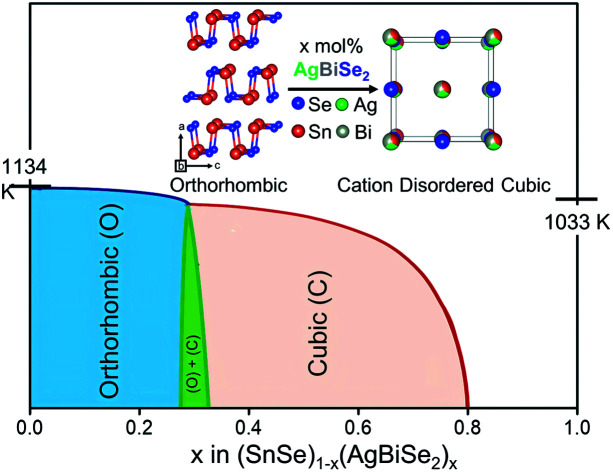

## Introduction

Thermoelectric materials can perform the conversion of waste heat into electricity and are believed to be the key to future energy management. In general, the performance of a thermoelectric material is determined by the dimensionless figure of merit, *zT*, which is given by *zT* = *S*^2^*σ*/*κ*, where *σ*, *S*, *κ* and *T* are the electrical conductivity, Seebeck coefficient, total thermal conductivity and absolute temperature, respectively.^1–3^ The total thermal conductivity is the sum of electrical thermal conductivity (*κ*_ele_) and lattice thermal conductivity (*κ*_lat_). In a typical degenerate semiconductor, all the above-mentioned parameters, except *κ*_lat_, are intertwined with each other in a trade-off fashion.^1^ Reduction in the *κ*_lat_ can be acquired by introducing crystal defects, alloying and nanostructuring,^4–9^ or by intrinsic phenomena.^10,11^ Amplification of the power factor (*S*^2^*σ*) is generally achieved by the improvement of the Seebeck coefficient through band convergence^12,13^ and resonance level formation.^14^ In addition, an enhancement of the electrical conductivity can be achieved through optimization of the carrier concentration by suitable doping.^12–15^ However, it remains a key challenge to optimally consolidate all these interdependent parameters in a single material to achieve high thermoelectric performance.

The persistent search for efficient thermoelectric materials in the last few years has been intense, systematic, and multipronged, leading to the discovery of new two-dimensional (2D) layered materials with significantly high thermoelectric performances.^16–18^ However, the extraordinary discovery of layered tin selenide (SnSe) from the group IV–VI metal chalcogenide family has stimulated huge excitement in the thermoelectric community.^19–30^ Single-crystals of p-type SnSe showed an unprecedented *zT* of 2.6 at 923 K along the crystallographic *b*-axis,^19^ whereas, n-type SnSe single crystals exhibited a record-high *zT* of 2.8 at 773 K along the *a*-axis.^21^ The superior thermoelectric performance of SnSe single crystals arises from its intrinsically ultralow lattice thermal conductivity (0.20 W m^−1^ K^−1^) that primarily originates from the presence of high anharmonicity and anisotropy in its crystal structure.^19,23^

SnSe crystalizes in three different structures: the stable orthorhombic (*Pnma*) phase at ambient temperature and pressure,^19,20^ the metastable orthorhombic (*Cmcm*) phase at high temperature,^23^ and the unstable rock-salt cubic (*Fm*3̄*m*) phase at high pressure.^31–33^ SnSe undergoes a displacive phase transition from the low-symmetry *Pnma* phase to the high-symmetry *Cmcm* phase at ∼800 K.^23^ This phase transition at elevated temperature results in extended defects and enhancement in the lattice strain, which degrade the mechanical properties of SnSe and limit its long-term power generation applications at high temperature.^34^ Furthermore, it has been reported that the high-symmetry cubic phase can only be stabilized at very high pressure or by inducing strain.^31–33,35^ However, the formation energy calculated from the density functional theory (DFT) for the orthorhombic *Pnma* and cubic *Fm*3̄*m* states are thermodynamically comparable.^31,36^ The cubic SnSe has been theoretically predicted to have large anharmonicity, high Seebeck coefficient and intrinsically low lattice thermal conductivity.^32,36^ Previously, cubic rock-salt SnSe was obtained in thin films grown on a Bi_2_Se_3_ substrate using molecular beam epitaxy;^37^ however, since it is a resource-consuming physical preparation process, an alternative method to prepare the cubic SnSe in bulk form is desired. Therefore, in this work, a chemical approach has been explored to stabilize the cubic rock-salt structure of SnSe and study the crystal and electronic structural evolution. Hitherto, the stabilization of cubic rock-salt polycrystalline SnSe has only been achieved *via* alloying with cubic rock salt AgSbSe_2_ and AgSbTe_2_.^34,36,38^ On the other hand, hexagonal AgBiSe_2_ has been successfully used to tune the carrier concentration and reduce the lattice thermal conductivity in the cubic phase of GeTe and GeSe systems.^39,40^ Hence, AgBiSe_2_ can be chosen as a suitable alloying material to tune the crystal structure, electrical and thermal properties of SnSe since it is isostructural to GeSe.

Herein, we have successfully stabilized the high-pressure cubic rock-salt phase of SnSe by alloying with AgBiSe_2_ (0.30 ≤ *x* ≤ 0.80) at ambient temperature and pressure. The solid solution mixing of AgBiSe_2_ with SnSe increases the configurational entropy by introducing atomic disorder into the system and consequently stabilizes the cubic phase under ambient conditions. The orthorhombic polycrystalline phase is stable in (SnSe)_1−*x*_(AgBiSe_2_)_*x*_ in the composition range of 0.00 ≤ *x* < 0.28, which corresponds to narrow band gap semiconductors. The band gap closes upon increasing the concentration of AgBiSe_2_ (0.30 ≤ *x* < 0.70) leading to the cubic rock-salt phase, which is consistent with the electronic structure calculated by DFT with the inclusion of spin orbit coupling. Interestingly, pristine cubic SnSe exhibits a topological crystalline insulator (TCI) quantum phase where the metallic surface states are protected by mirror symmetry, but the cubic (SnSe)_1−*x*_(AgBiSe_2_)_*x*_ (*x* = 0.33) possesses a semi-metallic electronic structure with overlapping conduction and valence bands. Cubic polycrystalline (SnSe)_1−*x*_(AgBiSe_2_)_*x*_ (*x* = 0.30) sample show n-type conduction at room temperature but the orthorhombic (SnSe)_1−*x*_(AgBiSe_2_)_*x*_ (0.00 ≤ *x* < 0.28) samples retain their p-type character. Orthorhombic (SnSe)_0.78_(AgBiSe_2_)_0.22_ exhibits superior electrical conductivity and power factors of ∼685 S cm^−1^ and ∼6.50 μW cm^−1^ K^−2^, respectively, at 823 K. As a result, a peak *zT* of 1.3 at 823 K has been obtained in the polycrystalline orthorhombic (SnSe)_0.78_(AgBiSe_2_)_0.22_ system. This study provides profound insight into the crystal and electric structural modulations and thermoelectric properties of polycrystalline (SnSe)_1−*x*_(AgBiSe_2_)_*x*_.

## Results and discussion

**Fig. 1:**
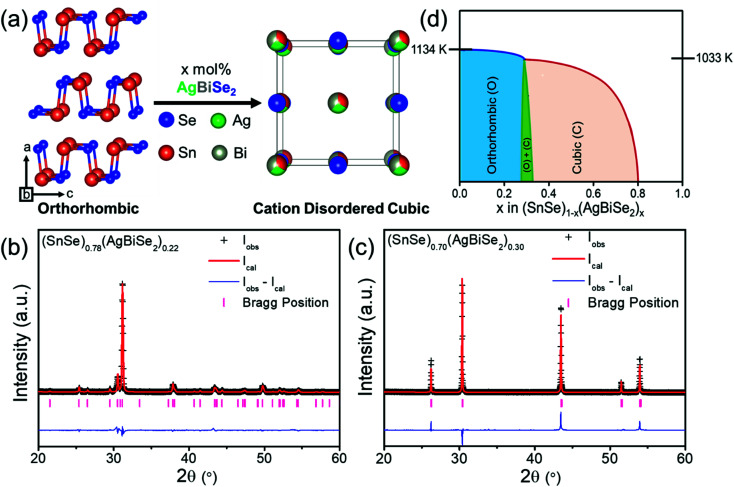
(a) Structural transformation of the orthorhombic SnSe into the rock-salt cubic structure upon AgBiSe_2_ alloying. Rietveld refinement of room-temperature PXRD data of (b) orthorhombic (SnSe)_0.78_(AgBiSe_2_)_0.22_ and (c) cubic (SnSe)_0.70_(AgBiSe_2_)_0.30_ samples. (d) A schematic phase diagram of the SnSe–AgBiSe_2_ system.

Polycrystalline (SnSe)_1−*x*_(AgBiSe_2_)_*x*_ (0.00 ≤ *x* ≤ 1.00) samples were synthesized in a vacuum-sealed tube reaction followed by ball milling and spark plasma sintering (SPS). Orthorhombic SnSe (*Pnma*), when alloyed with AgBiSe_2_, gradually transformed from orthorhombic to the face-centered cubic structure under ambient conditions. With the addition of 30 mol% AgBiSe_2_ in SnSe, a disordered rock-salt (*Fm*3̄*m*) phase was stabilized (Fig. 1a), which realized up to 80 mol% AgBiSe_2_ addition in SnSe. The room-temperature powder X-ray diffraction (PXRD) patterns of bulk (SnSe)_1−*x*_(AgBiSe_2_)_*x*_ samples can be indexed to the phase-pure orthorhombic phase when 0.00 ≤ *x* < 0.28, and to the face-centered cubic phase when 0.30 ≤ *x* ≤ 0.80 (Fig. S1, ESI†). On the contrary, the AgBiSe_2_-rich polycrystalline (SnSe)_1−*x*_(AgBiSe_2_)_*x*_ (when *x* > 0.80) samples resemble the room temperature hexagonal crystal structure of AgBiSe_2_ (space group *P*3̄*m*1). The Rietveld refinement of the room temperature PXRD patterns of orthorhombic (SnSe)_0.78_(AgBiSe_2_)_0.22_ and cubic (SnSe)_0.70_(AgBiSe_2_)_0.30_ are shown in Fig. 1b and c, respectively, which indicate that when AgBiSe_2_ is added to the SnSe system, the Ag and Bi atoms preferentially occupy the Sn sites and induce disorder (Tables S1 and S2, ESI†). This leads to the enhancement of the configurational entropy and the consequent stabilization of the high symmetry cubic phase under ambient conditions. A similar phenomenon can also be observed in other alloys where, the addition of extra elements can boost the total entropy of the system, resulting in the stabilization of higher symmetric phases.^39,41,42^

Field emission scanning electron microscopy (FESEM) in the backscattered electron mode (BSE) was performed on orthorhombic (SnSe)_0.78_(AgBiSe_2_)_0.22_ and cubic (SnSe)_0.70_(AgBiSe_2_)_0.30_ to check the purity of the polycrystals, which confirmed the absence of any secondary micro-precipitates (Fig. S2 and S3, ESI†) in both the samples. Energy dispersive X-ray (EDAX) elemental color mapping of Sn, Ag, Bi, and Se during FESEM for both the orthorhombic and cubic (SnSe)_1−*x*_(AgBiSe_2_)_*x*_ (Fig. S4 and S5, ESI†) confirmed that all the elements were uniformly distributed in the samples, indicating the homogeneity of the polycrystals.

The schematic phase diagram of the SnSe–AgBiSe_2_ system, as inferred from PXRD, DSC, and TGA analysis, is shown in Fig. 1d; the phase boundaries are, however, relative to the position of the orthorhombic, and cubic phases with respect to the AgBiSe_2_ concentration. Nevertheless, this schematic phase diagram clearly shows that the increase in the AgBiSe_2_ concentration in SnSe causes a structural phase evolution from the orthorhombic to the rock-salt crystal structure.

**Fig. 2:**
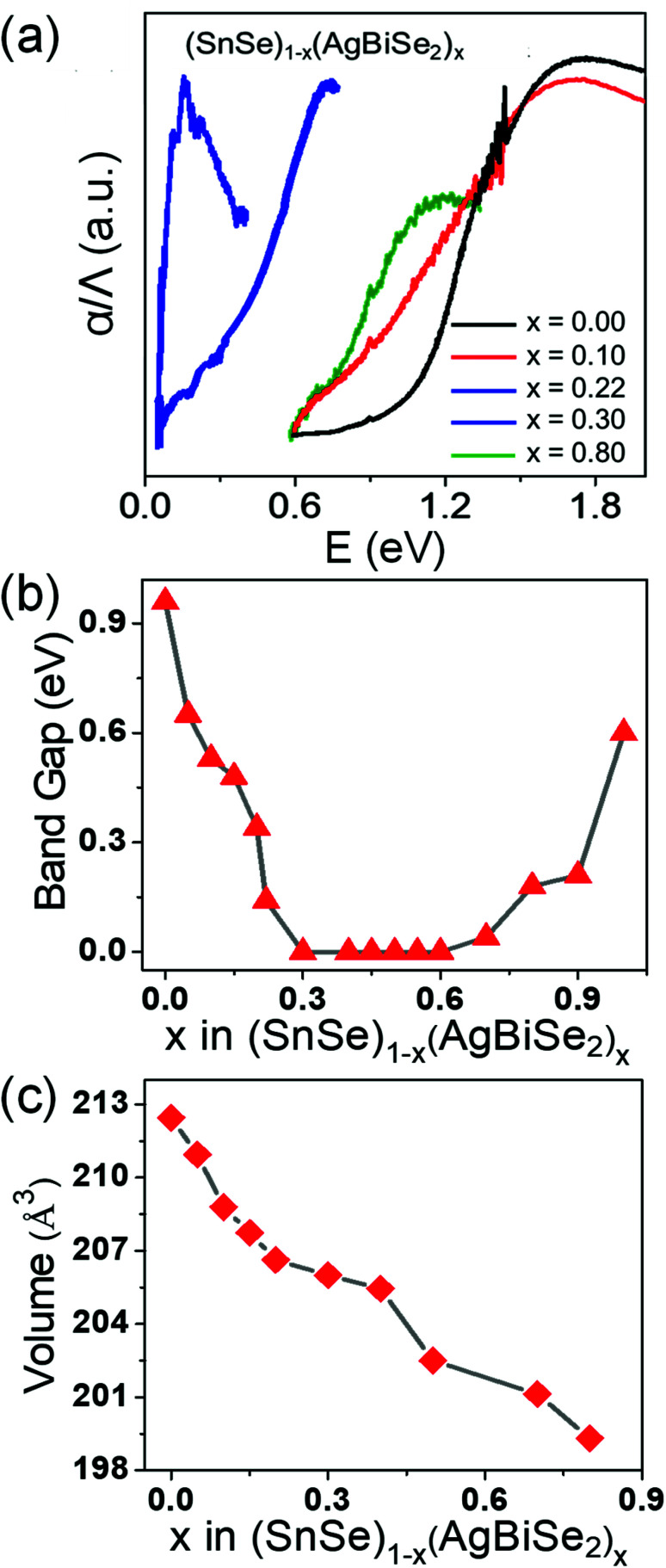
(a) Electronic absorption spectra, (b) band gap evolution and (c) unit cell volume of (SnSe)_1−*x*_(AgBiSe_2_)_*x*_ (0.00 ≤ *x* ≤ 0.80) as a function of AgBiSe_2_ concentration.

The band gap of the pristine orthorhombic SnSe was measured to be about 0.90 eV (Fig. 2a), which is consistent with previous literature reports.^19,20^ However, when SnSe was alloyed with AgBiSe_2_, the band gap closed rapidly near to zero at *x* = 0.30 (Fig. 2a and b) owing to the increase in the chemical pressure originating from a decrease in the unit cell volume from 213 Å^3^ (orthorhombic) to 206 Å^3^ (cubic) as shown in Fig. 2c. The band-gap evolution plot is shown in Fig. 2b, which depicts that upon increasing the concentration of AgBiSe_2_ above *x* = 0.24, the band gap closes. The probable reason could be the structural phase transition induced contraction of the unit cell volume, which increased the chemical pressure and consequently reduced the band gap.

**Fig. 3:**
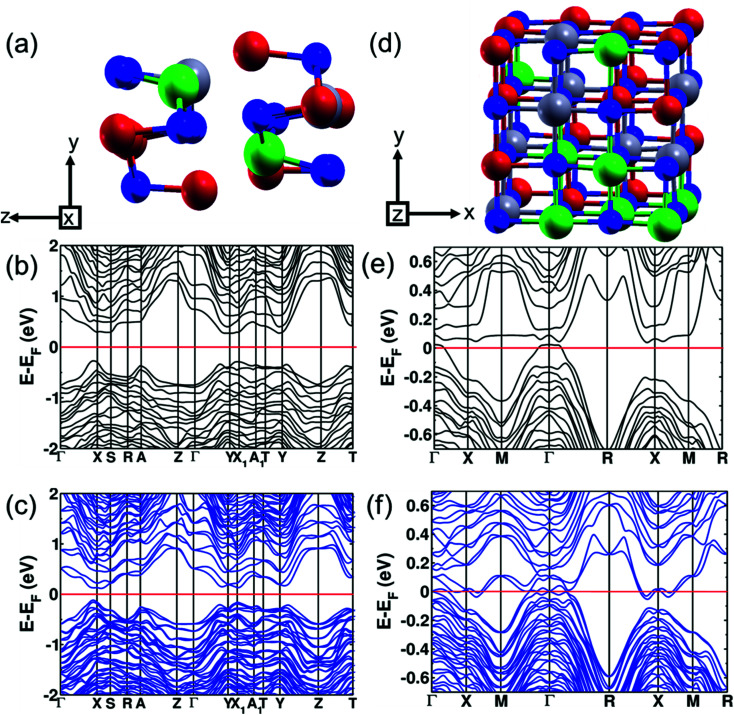
(a) SQS unit cell of modelled orthorhombic (SnSe)_0.80_(AgBiSe_2_)_0.20_ and its electronic structure calculated at the theoretical lattice constant (b) without the inclusion of the spin–orbit interaction (SOI) and (c) with SOI. (d) SQS unit cell of modelled cubic (SnSe)_0.67_(AgBiSe_2_)_0.33_. Electronic structure of cubic (SnSe)_0.67_(AgBiSe_2_)_0.33_ calculated at the theoretical lattice constant (e) without the inclusion of SOI and (f) with SOI. (Red, green, blue, and grey atoms represent Sn, Ag, Se and Bi, respectively).

**Fig. 4:**
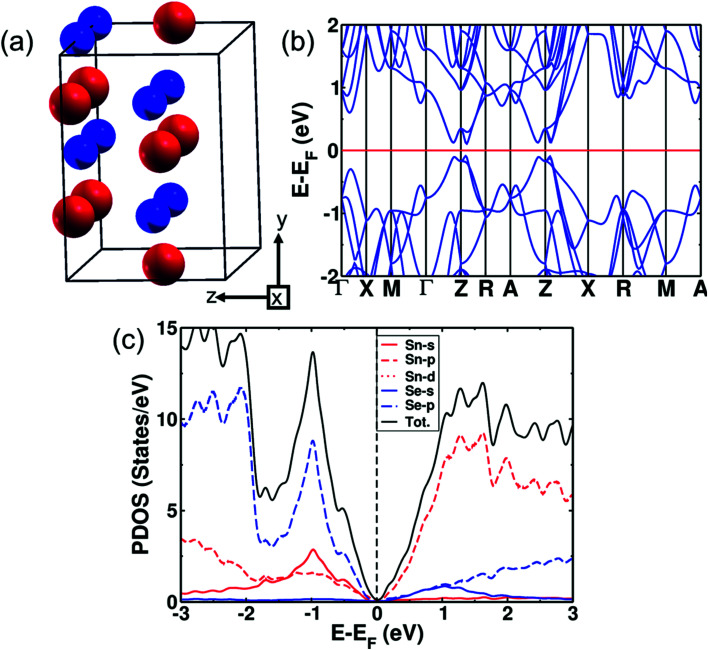
(a) Crystal structure of the supercell of cubic SnSe (Sn Red, Se blue). (b) Electronic structures of the √2 × √2 × 1 tetragonal supercell of the cubic structure of SnSe with the inclusion of the effects of spin–orbit coupling. (c) Electronic density of states (DOS) and projected density of states (PDOS) of cubic SnSe.

To study the evolution of the band gap with respect to alloying concentration, we used first-principles DFT calculations to determine the electronic band structure at three concentrations: pure SnSe (orthorhombic and cubic), (SnSe)_0.80_(AgBiSe_2_)_0.20_ (orthorhombic) and (SnSe)_0.67_(AgBiSe_2_)_0.33_ (cubic). The theoretical band gap of SnSe in the orthorhombic structure is 0.71 eV, with the inclusion of the spin–orbit coupling in calculations (Fig. S6, ESI†). Such underestimation of the band gap relative to the experimental band gap of 0.90 eV is typical of DFT calculations. We found that the theoretical band gap of SnSe decreased with AgBiSe_2_ alloying, consistent with the trend observed in experimental measurements using diffuse reflectance spectroscopy. The modelled structure of the orthorhombic (SnSe)_0.80_(AgBiSe_2_)_0.20_ generated using the special quasi-random structures (SQS) algorithm is shown in Fig. 3a. Our estimations of the band gap of (SnSe)_0.80_(AgBiSe_2_)_0.20_ are ∼0.51 eV and ∼0.24 eV without and with the inclusion of the spin–orbit coupling, respectively, as shown in Fig. 3b and c. The band gap obtained from calculation with spin orbit coupling is smaller than the experimentally observed value of ∼0.34 eV, as expected.

(SnSe)_1−*x*_(AgBiSe_2_)_*x*_ with *x* = 0.33 exists in the cubic phase (*Fm*3̄*m*). The electronic structure of pure cubic SnSe exhibited a band gap of 0.12 eV estimated with the inclusion of the spin–orbit coupling in calculations as shown in Fig. 4. Examination of the projected density of states (PDOS) (Fig. 4c) revealed that its valence band (VB) was contributed mostly by p orbitals of Se, and weakly by s and p orbitals of Sn. The conduction band (CB) was contributed primarily by the p orbitals of Sn. We determined the mirror Chern number (*n*_M_)^31,43^ and the *Z*_2_ topological invariant of pure cubic SnSe. *Z*_2_ characterizes the topological insulators (TI) (*Z*_2_ = 0 trivial insulator and *Z*_2_ = 1 nontrivial insulator) and the mirror Chern number is the topological invariant describing a topological crystalline insulator (TCI). In topological crystalline insulators (TCI),^43,44^ the gapless surface states are protected by mirror symmetry, in contrast to topological insulators where the time-reversal symmetry protects the surface states. The presence of mirror symmetry in the crystal structure of a material results in the presence of planes in the BZ giving rise to mirror symmetry-protected Dirac cones in the surface electronic structure. A TCI supports an even number of Dirac cones and band inversions in sharp contrast to a TI characterized by odd numbers of band inversions. TCIs are characterized by a non-zero mirror Chern number. The individual Chern numbers *C*_+i_ and *C*_−i_ are defined on a mirror-invariant plane for TCI. The mirror Chern number^43^ defined as *n*_M_ = (*C*_+i_ − *C*_−i_)/2 can be used as a topological invariant for TCI. The strong *Z*_2_ topological invariant (*ν*_0_) of the cubic SnSe is 0 (normal insulator) confirming its trivial electronic topology with respect to time reversal symmetry, while its *n*_M_ = 2 establishes its non-trivial band topology with respect to crystalline symmetry. Therefore, the cubic phase of SnSe is a TCI.

We have examined the electronic structure of cubic (SnSe)_1−*x*_(AgBiSe_2_)_*x*_ in two disordered configurations at *x* = 0.33 (Fig. S7a and S8a, ESI†) simulated with the model √2 × √2 × 1 supercell of the conventional cell of the *Fm*3̄*m* structure. The lower-symmetric disordered structure (Fig. S8a, ESI†) was simulated by interchanging a pair of Sn and Ag atoms in the first disordered structure (Fig. S7a, ESI†). The electronic structures in both cases showed a vanishing band gap with overlapping valence and conduction bands at the Fermi level, consistent with the trend observed in our experiments. In cubic SnSe (Fig. 4a), Sn occupies 4a (0.0, 0.0, 0.0) Wyckoff positions and Se occupies 4b (0.5, 0.5, 0.5) positions in the *Fm*3̄*m* space group. In the cubic phase of SnSe alloyed with 33 mol% AgBiSe_2_, the Ag and Bi atoms occupy any of the 4a (0, 0, 0) sites randomly; therefore, to take into account the effect of realistic chemical disorder we generated a model structure using the SQS algorithm. In the 2 × 2 × 2 supercell having 16 Sn, 8 Ag, 8 Bi and 32 Se atoms, 4b Wyckoff sites are occupied by Se atoms while Ag, Bi and Sn atoms randomly occupy 4a sites (Fig. 3d). Our estimation of the band gap of cubic (SnSe)_0.67_(AgBiSe_2_)_0.33_ calculated without spin–orbit coupling (Fig. 3e) is ∼0.07 eV, whereas the inclusion of the spin–orbit coupling closes this small gap and confirms its semi-metallic nature (Fig. 3f). For a deeper understanding of the electronic properties, we examined the projected density of states (PDOS) of the cubic phase of (SnSe)_0.67_(AgBiSe_2_)_0.33_ (Fig. S7c, ESI†). The valence band is dominated by the contribution from the p-orbitals of Se, similar to pure cubic SnSe, while the CB is contributed majorly by the p orbitals of Sn and along with additional new contributions from p-orbitals of Bi.

We calculated the relative stabilities of the orthorhombic and cubic phases of (SnSe)_1−*x*_(AgBiSe_2_)_*x*_ with varied *x* (where *x* = 0, 0.20, 0.33) to confirm the transformation of the orthorhombic SnSe (*Pnma*) into the face-centered cubic structure with AgBiSe_2_ alloying. Here, we used the same SQS supercell in the calculation of cubic and orthorhombic structures at each value of *x* by deforming and distorting the cubic structure to the orthorhombic structure and relaxing it further. For pristine SnSe, the orthorhombic phase is lower in energy with respect to its cubic phase by 2 meV f.u.^−1^ confirming its greater stability as seen in the experimental results. At 20% AgBiSe_2_ alloying, the orthorhombic phase remains lower in energy by ∼46 meV f.u.^−1^ with respect to its cubic phase, consistent with the experimentally observed orthorhombic structure of (SnSe)_0.80_(AgBiSe_2_)_0.20_. Our estimation of the energy difference between the orthorhombic and cubic (SnSe)_0.67_(AgBiSe_2_)_0.33_ was 1620 meV f.u.^−1^, stabilizing the cubic phase over its orthorhombic phase, confirming that the rock-salt (*Fm*3̄*m*) phase is stabilized with the addition of 30 mol% AgBiSe_2_ in SnSe.

Thermoelectric properties were measured for the ball-milled and SPS-processed anisotropic orthorhombic and isotropic cubic polycrystalline (SnSe)_1−*x*_(AgBiSe_2_)_*x*_ (0.00 ≤ *x* ≤ 0.30) samples. The cubic polycrystalline (SnSe)_0.70_(AgBiSe_2_)_0.30_ sample showed lower thermoelectric performance as compared to the orthorhombic (SnSe)_0.78_(AgBiSe_2_)_0.22_. On the other hand, improved thermoelectric performance was observed in the anisotropic orthorhombic phase when measurements were carried out parallel to the SPS pressing direction (Fig. S9, ESI†).

**Fig. 5:**
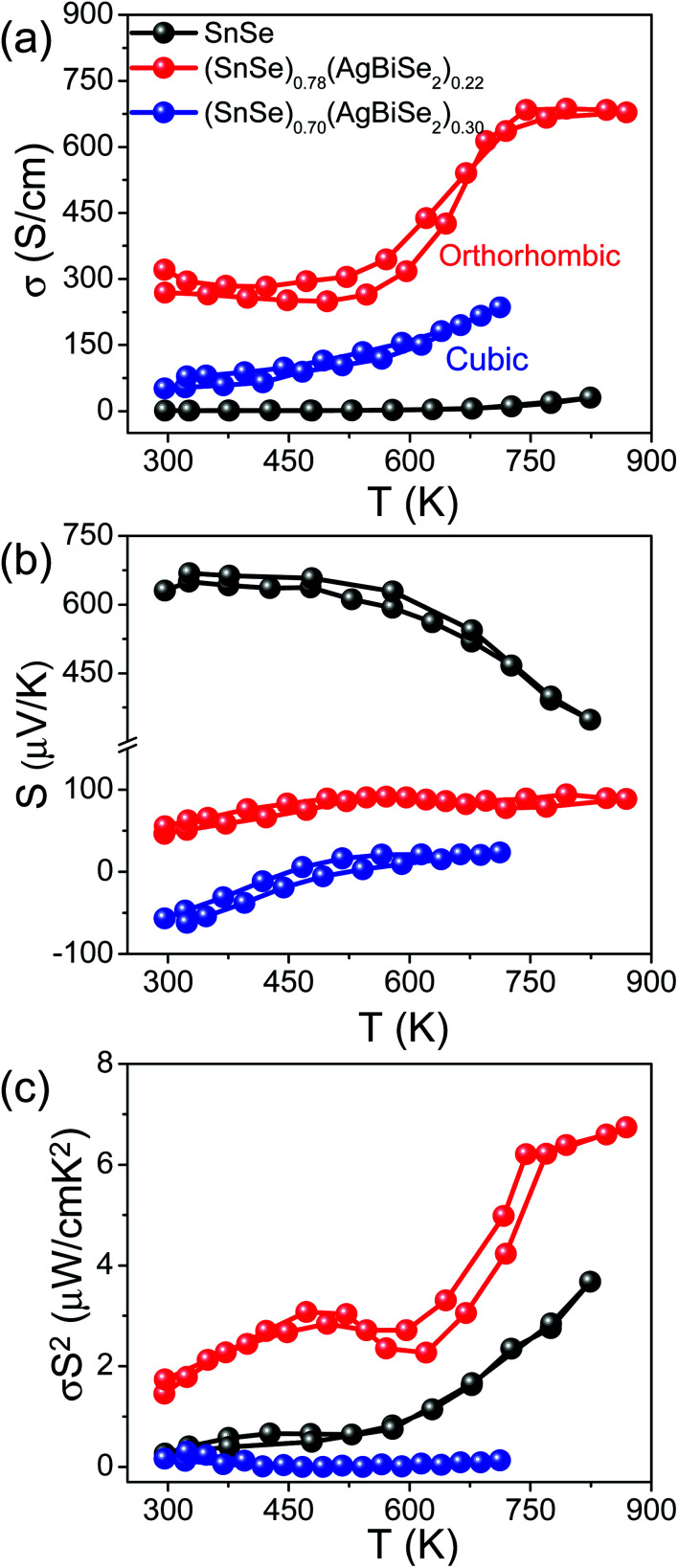
Temperature-dependent (a) electrical conductivity (*σ*), (b) Seebeck coefficient (*S*) and (c) power factor (*S*^2^*σ*) of ball-milled polycrystalline (SnSe)_1−*x*_(AgBiSe_2_)_*x*_ (where, *x* = 0, 0.22 are orthorhombic and *x* = 0.30 is cubic in nature) samples measured parallel to the SPS pressing direction.

Fig. 5 presents the thermoelectric properties of (SnSe)_1−*x*_(AgBiSe_2_)_*x*_ (0.00 ≤ *x* ≤ 0.30) measured parallel to the SPS pressing direction. Fig. 5a depicts the temperature-dependent electrical conductivity of the pristine orthorhombic SnSe, orthorhombic (SnSe)_0.78_(AgBiSe_2_)_0.22_ and cubic (SnSe)_0.70_(AgBiSe_2_)_0.30._ The *σ* of the pristine orthorhombic SnSe was 1.18 S cm^−1^ at 300 K, which increased to about 270 S cm^−1^ upon 22 mol% AgBiSe_2_ alloying at room temperature. For all the (SnSe)_1−*x*_(AgBiSe_2_)_*x*_ (0.00 ≤ *x* < 0.28) samples, electrical conductivity increased with increasing temperature, which indicated the semiconducting transport (Fig. 5a). The sharp increase in electrical conductivity at 610 K for orthorhombic (SnSe)_0.78_(AgBiSe_2_)_0.22_ is an indication of the phase transition from lower symmetric *Pnma* to the higher symmetric *Cmcm* phase.^24,45^ The solid-solution mixing of AgBiSe_2_ with SnSe significantly increased *σ* due to the enhancement in the carrier concentration from 8.9 × 10^17^ cm^−3^ for pristine SnSe to 8.2 × 10^19^ cm^−3^ in (SnSe)_0.78_(AgBiSe_2_)_0.22_. A positive value of the Hall coefficient indicated that holes are the majority carriers in orthorhombic (SnSe)_1−*x*_(AgBiSe_2_)_*x*_ (0.00 ≤ *x* < 0.28) making it a p-type semiconductor (Table S3, ESI†). Further, on addition of 30 mol% AgBiSe_2_, the sample became n-type in nature as confirmed from the negative sign of the Hall coefficient and the room temperature carrier concentration was 6.17 × 10^18^ cm^−3^ for the cubic (SnSe)_0.70_(AgBiSe_2_)_0.30_.

The measured value of the Seebeck coefficient of the pristine orthorhombic SnSe was 631 μV K^−1^ at 300 K (Fig. 5b), which decreased to 46 μV K^−1^ for orthorhombic (SnSe)_0.78_(AgBiSe_2_)_0.22_ and with a further increase in the AgBiSe_2_ concentration, the sample showed n-type conduction with an *S* value of −57 μV K^−1^ for (SnSe)_0.70_(AgBiSe_2_)_0.30_. The lower value of the Seebeck coefficient was consistent with the high carrier concentration in these samples. However, the cubic (SnSe)_0.70_(AgBiSe_2_)_0.30_ showed an anomalous trend in the *S* values arising mainly due to the onset of bipolar conduction. Due to the optimization in carrier concentration and electrical conductivity, a moderate power factor of ∼6.52 μW cm^−1^ K^−2^ was achieved in the (SnSe)_0.78_(AgBiSe_2_)_0.22_ sample at 873 K (Fig. 5c).

**Fig. 6:**
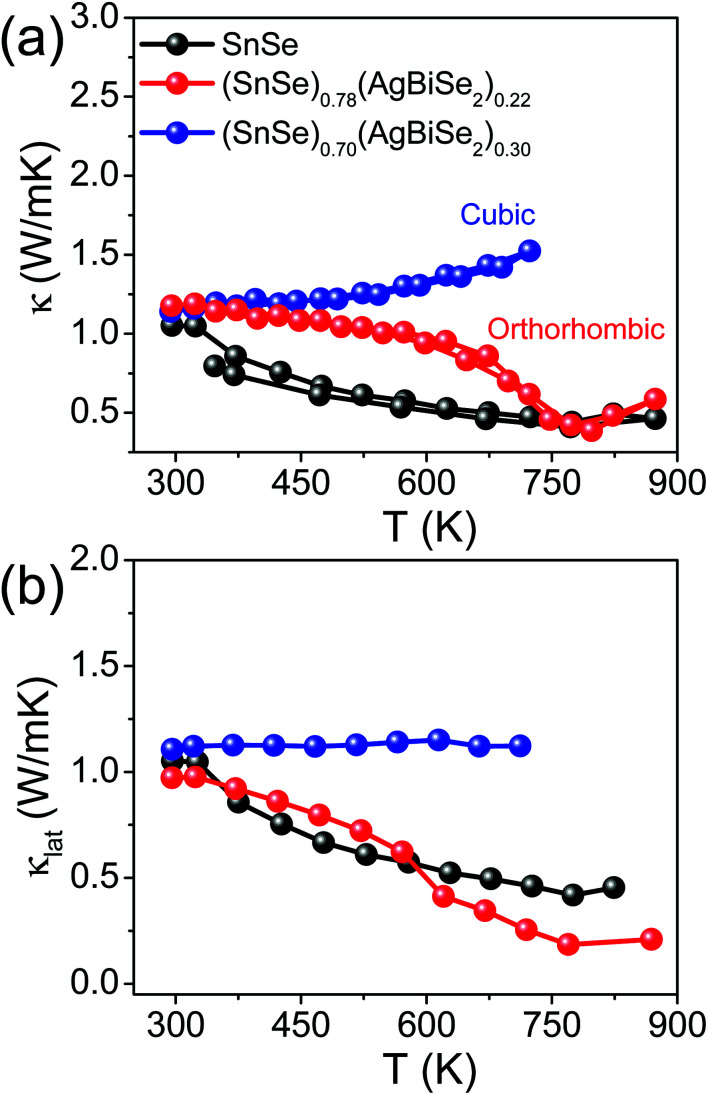
Temperature-dependent (a) total thermal conductivity (*κ*), and (b) lattice thermal conductivity (*κ*_lat_) of polycrystalline (SnSe)_1−*x*_(AgBiSe_2_)_*x*_ (where, *x* = 0, 0.22 are orthorhombic and *x* = 0.30 is cubic) samples measured parallel to the SPS pressing direction.

**Fig. 7:**
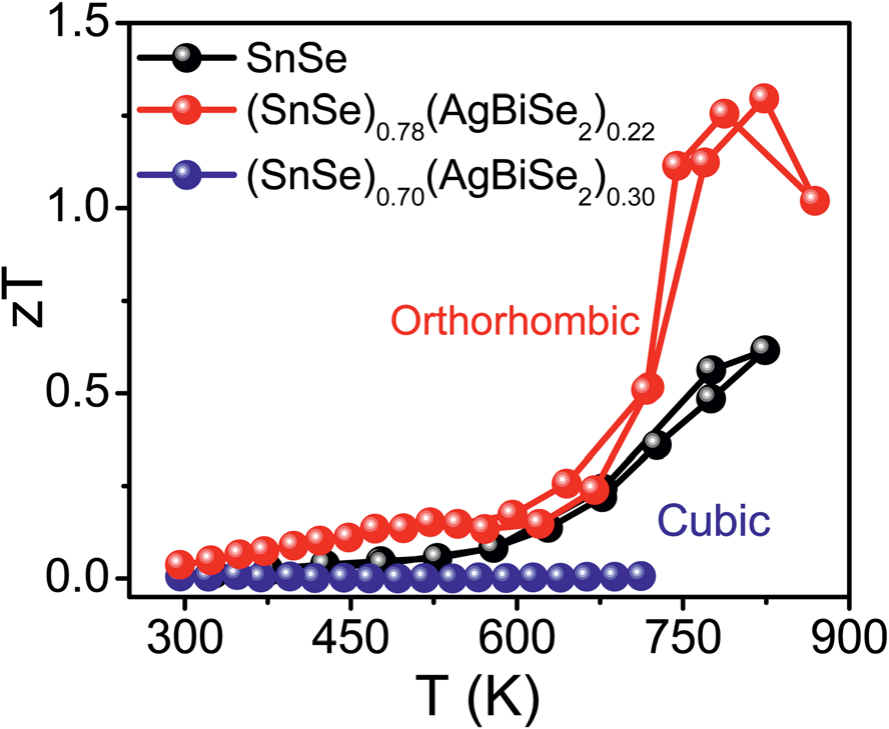
Temperature-dependent thermoelectric figure of merit (*zT*) of polycrystalline (SnSe)_1−*x*_(AgBiSe_2_)_*x*_ (where, *x* = 0, 0.22 are orthorhombic and *x* = 0.30 is cubic) samples measured parallel to the SPS pressing direction.

The total thermal conductivity of the pristine orthorhombic SnSe, orthorhombic (SnSe)_0.78_(AgBiSe_2_)_0.22_ and cubic (SnSe)_0.70_(AgBiSe_2_)_0.30_ measured parallel to the SPS pressing direction are presented in Fig. 6a. However, the room temperature total thermal conductivity is higher for the (SnSe)_1−*x*_(AgBiSe_2_)_*x*_ (*x* = 0.22 and 0.30) samples as compared to the pristine SnSe, which arises mainly due to the greater contributions from the electrical thermal conductivity (Fig. S10, ESI†). Ball-milled and SPS-processed pristine SnSe exhibited a lattice thermal conductivity (*κ*_lat_) of 1.08 W m^−1^ K^−1^ at 300 K, which decreased to 0.43 W m^−1^ K^−1^ at 773 K (Fig. 6b). When SnSe was alloyed with AgBiSe_2_, *κ*_lat_ in the cubic (SnSe)_0.70_(AgBiSe_2_)_0.30_ increased significantly to ∼1.13 W m^−1^ K^−1^ at 300 K (Fig. 6b). The low lattice thermal conductivity in orthorhombic SnSe was mainly caused by the damping phonon vibrations due to the presence of significant lattice anharmonicity.^19,20^ However, in the as-prepared cubic (SnSe)_0.70_(AgBiSe_2_)_0.30_, the higher lattice thermal conductivity can be attributed to the destruction of the layered orthorhombic crystal structure of SnSe. The orthorhombic (SnSe)_0.78_(AgBiSe_2_)_0.22_ sample showed an ultralow lattice thermal conductivity of ∼0.19 W m^−1^ K^−1^ at 773 K due to the enhanced phonon scattering induced by the point defects due to the entropy-driven solid solution in the SnSe–AgBiSe_2_ system in addition to lattice anharmonicity. The ultra-low *κ*_lat_ of (SnSe)_0.78_(AgBiSe_2_)_0.22_ at high temperature is comparable to that of the other high-performance SnSe-based polycrystalline samples (Table S5, ESI†). Thus, as a collective result of improved carrier concentration, elevated power factor and low thermal conductivity, a high *zT* of 1.3 has been achieved in the polycrystalline orthorhombic (SnSe)_0.78_(AgBiSe_2_)_0.22_ sample at 823 K (Fig. 7) when measured parallel to the SPS pressing direction, which is reversible and reproducible for different batches of samples, as well as for heating–cooling cycles (Fig. S11, ESI†).

## Conclusions

The electronic and crystal structures of SnSe have been tailored with the addition of AgBiSe_2_. The layered orthorhombic phase is stable in (SnSe)_1−*x*_(AgBiSe_2_)_*x*_ in the composition range of 0.00 ≤ *x* < 0.28, which corresponds to narrow band gap semiconductors. The high-pressure cubic rock-salt phase of SnSe has been stabilized under ambient conditions with increasing AgBiSe_2_ concentration to 0.30 ≤ *x* ≤ 0.80. Solid solution mixing of AgBiSe_2_ with SnSe increased the configurational entropy by introducing atomic disorder into the system and consequently stabilized the cubic phase under ambient conditions. The anomalous closing of the band gap of SnSe with increasing AgBiSe_2_ concentration was caused by the influence of the increase in chemical pressure. Electronic structures of the orthorhombic and cubic phases of (SnSe)_1−*x*_(AgBiSe_2_)_*x*_ showed the emergence of the band gap at higher AgBiSe_2_ concentrations in SnSe. The pristine cubic SnSe exhibited a topological crystalline insulator (TCI) phase but the cubic (SnSe)_1−*x*_(AgBiSe_2_) (*x* = 0.33) showed a semi-metallic electronic structure with overlapping conduction and valence bands. While the cubic (SnSe)_0.70_(AgBiSe_2_)_0.30_ sample showed n-type conduction, the orthorhombic (SnSe)_0.78_(AgBiSe_2_)_0.22_ retained the p-type nature at room temperature. We have achieved a high *zT* of 1.3 in the p-type polycrystalline orthorhombic (SnSe)_0.78_(AgBiSe_2_)_0.22_ at 823 K *via* the optimization of the carrier concentration and electronic properties.

## Data availability

All data are available in the manuscript or in the ESI.†

## Author contributions

K. B. conceived the idea and designed the study. S. C. and K. B. carried out the synthesis, characterizations, thermoelectric experiments and analysis of data. R. A. and U. V. W. carried out the theoretical calculations. All authors contributed to writing the manuscript.

## Conflicts of interest

The authors declare no competing financial interest.

## Supplementary Material

SC-012-D1SC03696C-s001
