# Supplementary material for: Modulation of the electronic structure and thermoelectric properties of orthorhombic and cubic SnSe by AgBiSe2 alloying
Source: Chem Sci. 2021 Aug 31;12(39):13074–82. doi: 10.1039/d1sc03696c (PMC8513838; doi:10.1039/d1sc03696c)
Supplement: SC-012-D1SC03696C-s001 [file SC-012-D1SC03696C-s001.pdf]

This journal is © The Royal Society of Chemistry 2021

Modulation of Electronic Structure and Thermoelectric Properties of Orthorhombic and Cubic SnSe by $AgBiSe_2$ Alloying

Sushmita Chandra[1,4], Raagya Arora[2,3], Umesh V. Waghmare[3,4] and Kanishka Biswas[*,1,4]

[1]*New Chemistry Unit,* [2]*Chemistry and Physics of Materials Unit and* [3]*Theoretical Sciences Unit,* [4]*School of Advanced Materials and International Centre for Materials, Jawaharlal Nehru Centre for Advanced Scientific Research (JNCASR), Jakkur P.O., Bangalore 560064, India*

**Email: kanishka@jncasr.ac.in*

METHODS

Reagents. The high purity elements utilized for the synthesis of $(SnSe)_{1-x}(AgBiSe_2)_x$ ($0.00 \le x \le 1.00$) samples are tin (Alfa Aesar 99.99+ %), silver (Ag, Aldrich 99.999%), bismuth (Alfa Aesar 99.9999%), and selenium (Se, Alfa Aesar 99.9999%).

Synthesis. Polycrystalline $(SnSe)_{1-x}(AgBiSe_2)_x$ ($0.00 \le x \le 1.00$) samples were synthesized by combining stoichiometric ratios of elemental Ag, Bi, Sn and Se in high quality quartz tubes. The quartz tubes were maintained at a pressure of 10^{-5} Torr and sealed under vacuum. The seal tubes were initially heated to 773 K over a period of 12 hrs, then heated again to 1223 K in next 5 hrs followed by annealing for 10 hrs and subsequently cooled to room temperature for a period of 15 hrs. The resulted ingots were crushed by using a mortar and pestle and sieved to fine powder. After that, ball-milling has been carried out at a speed of 250 rpm for 4 hrs at N_2 atmosphere in stainless-steel containers using a planetary Ball Mill (FRITSCH PULVERISETTE 7, Germany).

Spark plasma sintering (SPS). SPS was done using a SPS211-LX (Dr. Sinter Lab) instrument. The finely powdered samples were sintered to prepare a cylinder (10 mm × 8 mm) using graphite dies at 40 MPa pressure and 450 °C temperature. The samples were cut and polished

in different directions to measure the anisotropic electrical and thermal transport properties of $(SnSe)_{1-x}(AgBiSe_2)_x$ ($0.00 \leq x \leq 0.80$).

Powder X-ray diffraction. Room temperature powder X-ray diffraction for all the samples were recorded using a Cu K_α ($\lambda = 1.5406$ Å) radiation on a Bruker D8 Diffractometer. Rietveld refinement of the PXRD pattern was performed using FULLPROF software.

Field emission scanning electron microscopy (FESEM) in back-scattered electron (BSE) mode. FESEM-BSE images were taken using ZEISS Gemini SEM – Field Emission Scanning Electron Microscope.

Band gap measurement. To estimate optical band gap of the as-synthesized specimens of $(SnSe)_{1-x}(AgBiSe_2)_x$ ($0.00 \leq x \leq 1.00$), diffuse reflectance measurements were carried out with finely grounded powder at room temperature using a Perkin-Elmer Lambda 900 UV/Vis/near-IR spectrometer in reflectance mode (λ = 2500-250 nm) and FT-IR Bruker IFS 66V/S spectrometer (λ = 4000-400 cm^{-1}), respectively. Absorption (α/Λ) data were calculated from the reflectance data using Kubelka-Munk equation: $\alpha/\Lambda = (1-R)^2/(2R)$, where R is the reflectance, α and Λ are respectively the absorption and scattering coefficients. The energy band gap was then determined from α/Λ vs. E (eV) plot.

Electrical transport. Electrical conductivity and Seebeck coefficients were measured simultaneously under helium atmosphere from room temperature to 850 K on a ULVAC-RIKO ZEM-3 instrument system. The SPS processed sample were cut and polished in a rectangular shape with the dimensions of ~ 2 × 2 × 8 mm^3 to carry out the measurements. Electrical and thermal transport were measured in same direction.

Hall measurement. For determining the carrier concentrations, Hall measurements were carried out on the same rectangular specimens used for electrical transport measurement in four-contact geometry up to a magnetic field of 0.57 T at room-temperature using custom-built equipment developed by Excel Instruments.

Thermal transport. Temperature dependent thermal diffusivity (*D*) was evaluated using a laser flash diffusivity technique in a Netzsch LFA-457 instrument. In addition, temperature dependent heat capacity was also measured in the same instrument by using a standard pyroceram (Fig. S12). Next, the total thermal conductivity (κ) was derived using the formula, $\kappa = D.C_p.\rho$, where ρ is density of the sample and the experimentally determined density was found to be ~97% of the theoretical density. Further, the electrical thermal conductivity, κ_{ele} were derived using Wiedemann-Franz Law, $\kappa_{ele} = L\sigma T$, where L denotes the Lorenz number which was estimated by fitting the temperature dependent Seebeck data[1–3] and provided in Fig. S13.

Computational details. Our first-principles calculations within density functional theory (DFT) were performed with QUANTUM ESPRESSO Package (QE) and projector augmented wave (PAW) potentials.[4] Electronic exchange and correlation energy was treated within a generalized gradient approximated (GGA)[5] functional with Perdew, Burke, and Ernzerhof (PBE) parametrization.[6]

Electronic wave functions and charge density were represented using plane wave basis sets truncated at cut-off energies of 45 Ry and 360 Ry respectively. The discontinuity in occupation numbers of electronic states was smeared with broadening temperature of k_BT = 0.003 Ry in a Fermi-Dirac distribution function. We determined electronic structure of $(SnSe)_{1-x}(AgBiSe_2)_x$ in crystal structures with optimized (minimum energy) lattice parameters. At ambient conditions, SnSe stabilizes in the orthorhombic *Pnma* phase containing eight atoms in the periodic unit cell. Integrations over its Brillouin Zone (BZ) were sampled on a uniform 8×8×8 mesh of k-points. Electronic spectrum was determined at Bloch vectors along high symmetry lines (X -Γ - Y - P - Γ – A - Z – Γ - T) in the BZS. Our optimized lattice parameters for pristine SnSe in the orthorhombic structure (*Pnma*) are a = 11.77 Å, b = 4.22 Å, c = 4.53 Å, which are within the typical GGA errors of experimental lattice parameters (a = 11.57 Å, b

= 4.19 Å, c = 4.46 Å). The cubic phase of $(SnSe)_{0.67}(AgBiSe_2)_{0.33}$ was simulated using a √2x√2x1 supercell of conventional $Fm\bar{3}m$ structure containing 16 atoms. Our estimates of lattice parameters of $(SnSe)_{0.67}(AgBiSe_2)_{0.33}$ in this structure (√2x√2x1 supercell) are a = 8.43Å, b = 8.43Å, c = 5.96Å, which are within the typical GGA errors of experimental values of a = 8.36Å, b = 8.36Å, c = 5.91Å. Electronic spectrum was determined at Bloch vectors along high symmetry lines (Γ - X - M - Γ - Z - R - A - Z - X - R - M - A) in the BZ of its tetragonal unit by including the spin-orbit coupling (SOC) in calculations with fully relativistic potentials.[7]

The special quasirandom structures (SQS) of $(SnSe)_{0.67}(AgBiSe_2)_{0.33}$ in the cubic phase and of $(SnSe)_{0.8}(AgBiSe_2)_{0.2}$ in the orthorhombic phase were generated using the Monte Carlo SQS tool in the Alloy Theoretical Automated Toolkit (ATAT).[8] Our estimates of lattice parameters of the cubic structure of $(SnSe)_{0.67}(AgBiSe_2)_{0.33}$ are a = b = c = 5.98 Å, and of orthorhombic $(SnSe)_{0.8}(AgBiSe_2)_{0.2}$ are a = 12.05 Å, b = 4.26 Å and c = 4.52 Å. To determine the bulk electronic topology of cubic SnSe, we used Z2PACKcode[9] to calculate the Z_2 topological invariants and mirror Chern number (n_M). This code uses hybrid Wannier functions[10,11] and employs the ideas of time-reversal polarization in determination of the Z_2 invariants.

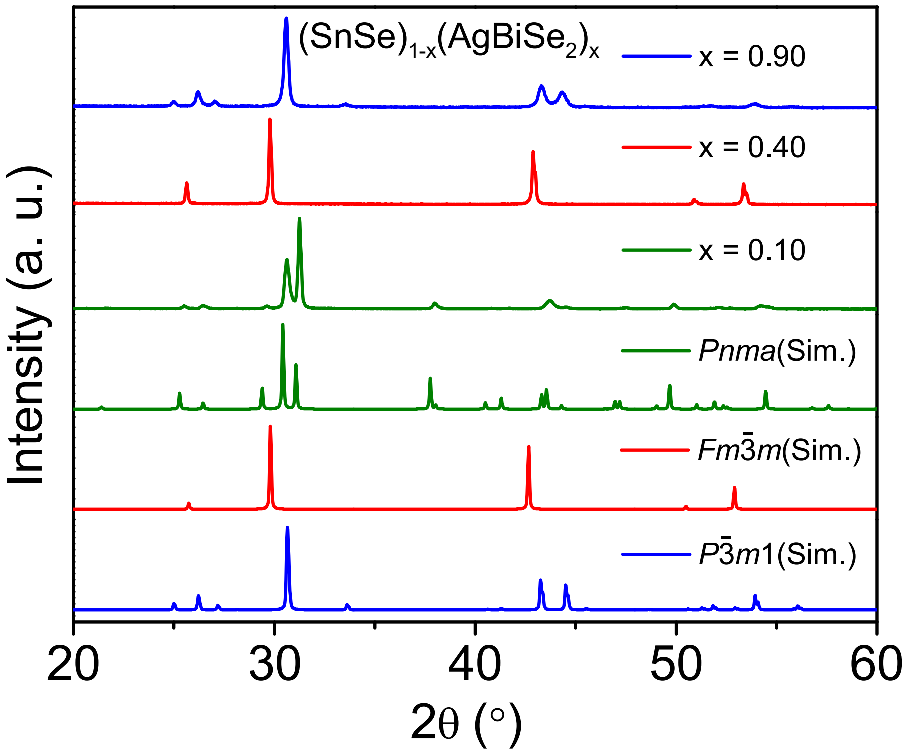

Fig. S1. PXRD patterns of polycrystalline (SnSe)$_{1-x}$(AgBiSe$_2$)$_x$ samples where the composition ranging in between $0.00 \leq x < 0.28$ are orthorhombic, $0.30 \leq x \leq 0.80$ are cubic and $0.80 < x \leq 1.00$ are hexagonal.

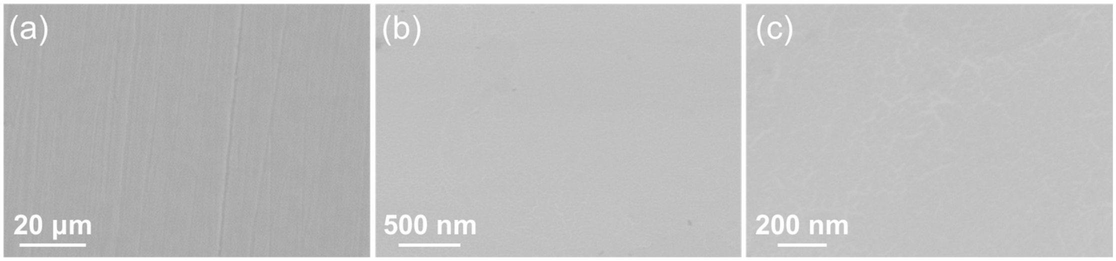

Fig. S2. FESEM-BSE images for ball milled and SPS processed orthorhombic (SnSe)$_{0.78}$(AgBiSe$_2$)$_{0.22}$ polycrystal with different resolution. The line like features present in (a) are due to polishing of sample.

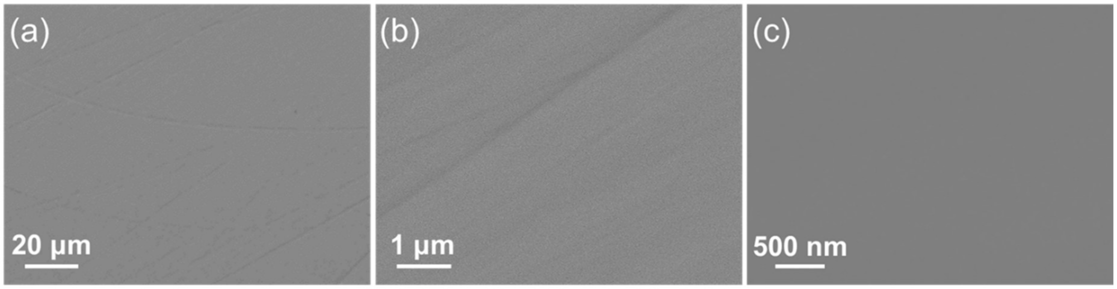

Fig. S3. FESEM-BSE images for ball milled and SPS processed cubic $(SnSe)_{0.70}(AgBiSe_2)_{0.30}$ polycrystal with different resolution. The line like features present in (a) & (b) are due to polishing of sample.

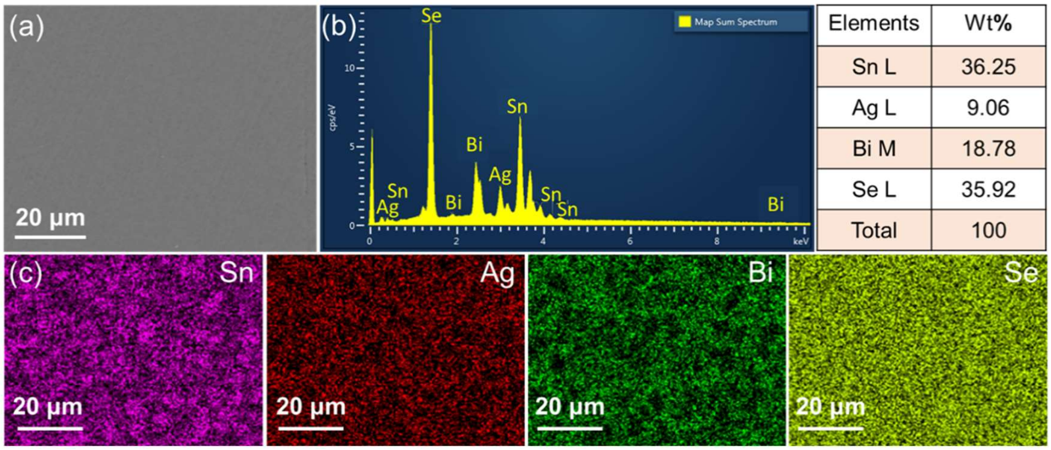

Fig. S4. (a) Backscattered electron images taken during FESEM for ball milled and SPS processed orthorhombic $(SnSe)_{0.78}(AgBiSe_2)_{0.22}$ polycrystal with corresponding EDAX spectra in (b). (c) EDAX elemental color mapping for Sn, Ag, Bi and Se for the area in (a).

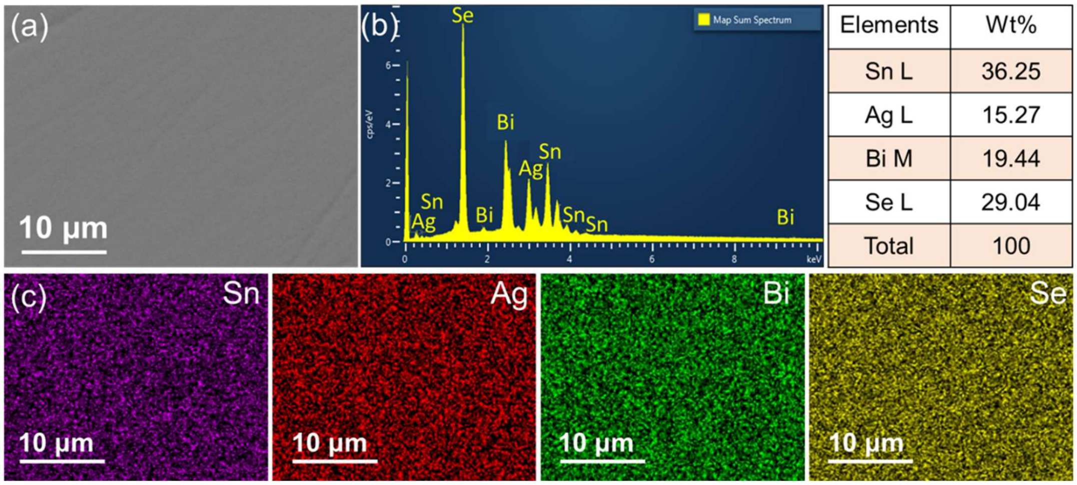

Fig. S5. (a) Backscattered electron images taken during FESEM for ball milled and SPS processed cubic $(SnSe)_{0.70}(AgBiSe_2)_{0.30}$ polycrystal with corresponding EDAX spectra in (b). (c) EDAX elemental color mapping for Sn, Ag, Bi and Se for the area in (a).

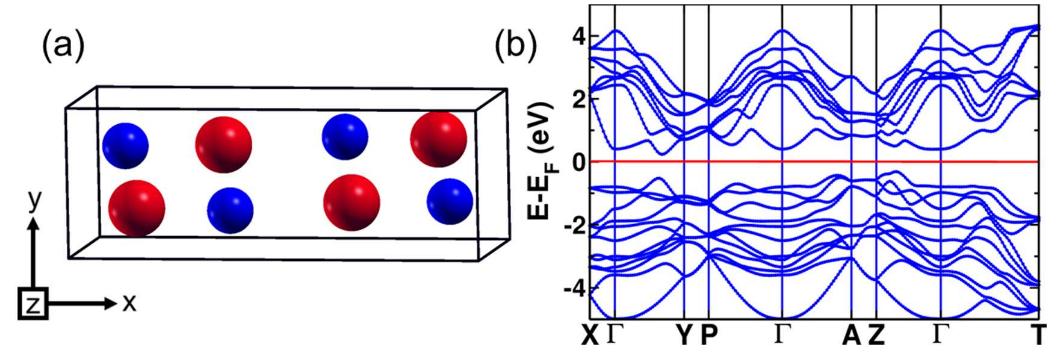

Fig. S6. (a) Crystal structure of SnSe in the orthorhombic unit cell with space group *Pnma* (Sn Red, Se blue). (b) Electronic structures of the orthorhombic (*Pnma*) SnSe with the inclusion of the effect of spin-orbit coupling.

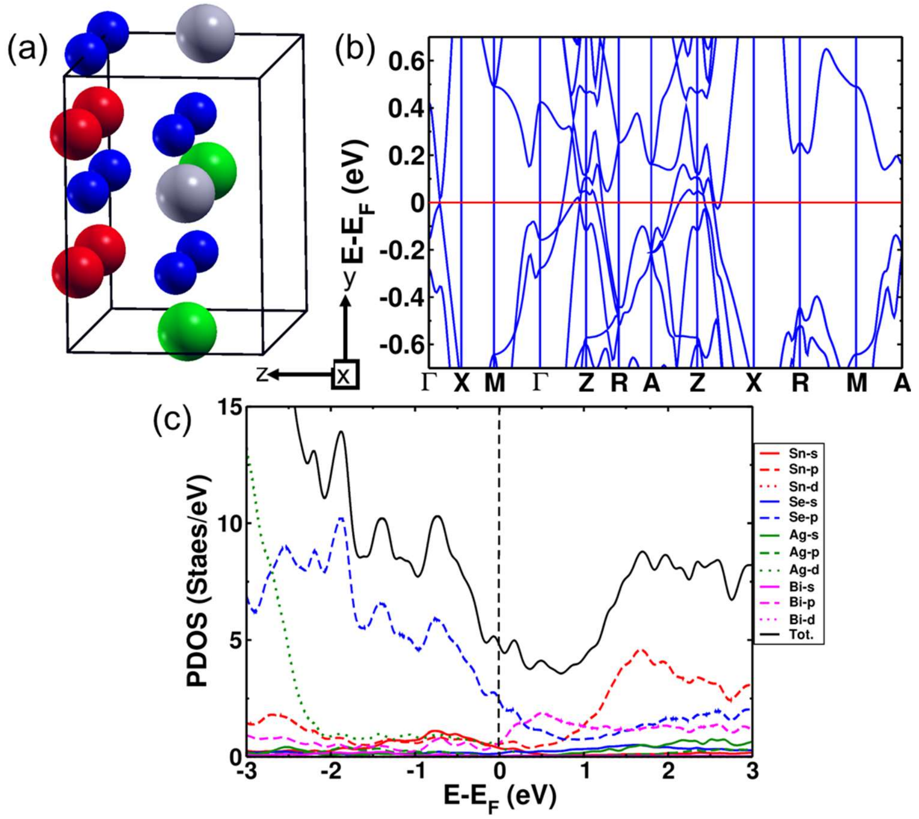

Fig. S7. (a) Crystal structure of disordered cubic $(SnSe)_{0.67}(AgBiSe_2)_{0.33}$ (Sn Red, Se blue, Ag green, Bi grey). (b) Electronic structures of the √2×√2×1 tetragonal supercell of the cubic phase of $(SnSe)_{0.67}(AgBiSe_2)_{0.33}$ with the inclusion of the effect of spin-orbit coupling. (c) Electronic density of states (DOS) and projected density of states (PDOS) of cubic $(SnSe)_{0.67}(AgBiSe2)_{0.33}$.

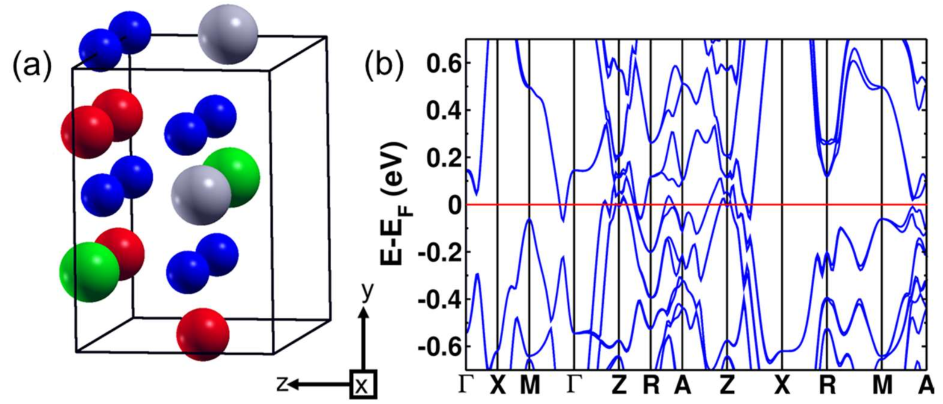

Fig. S8. (a) Crystal structure of lower-symmetric disordered cubic $(SnSe)_{0.67}(AgBiSe_2)_{0.33}$ (Sn Red, Se blue, Ag green, Bi grey) obtained by interchanging a pair or Sn and Ag atoms in Fig. S3a. (b) Electronic structure of the second configuration of √2×√2×1 tetragonal supercell of cubic structure of $(SnSe)_{0.67}(AgBiSe_2)_{0.33}$ with the inclusion of the effect of spin-orbit coupling.

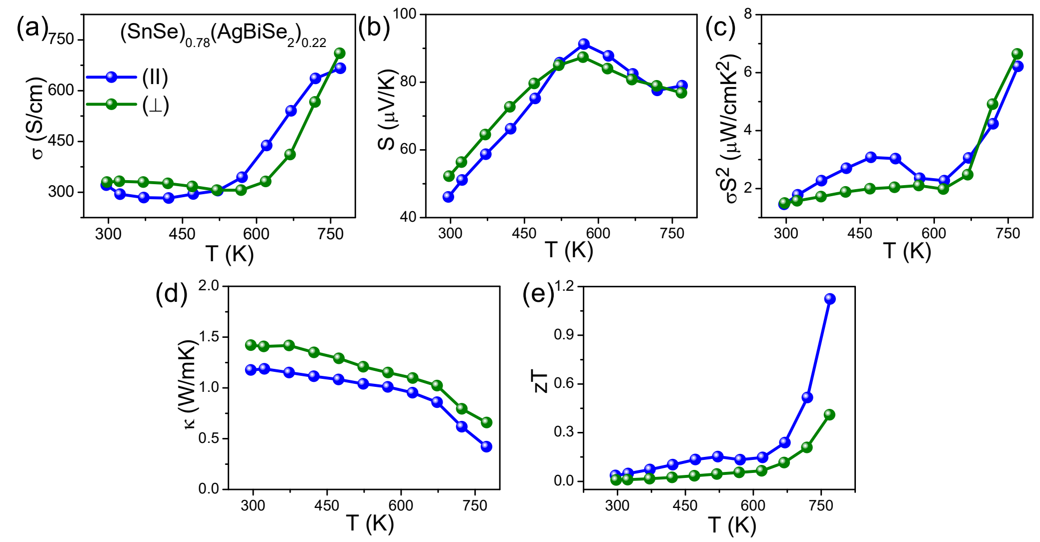

Fig. S9. Temperature dependent (a) electrical conductivity (σ), (b) Seebeck coefficient (S), (c) power factor ($S^2\sigma$), (d) total thermal conductivity (κ), and (e) thermoelectric figure of merit (zT) of ball milled and SPS processed orthorhombic $(SnSe)_{0.78}(AgBiSe_2)_{0.22}$ sample measured along both parallel and perpendicular to SPS pressing directions.

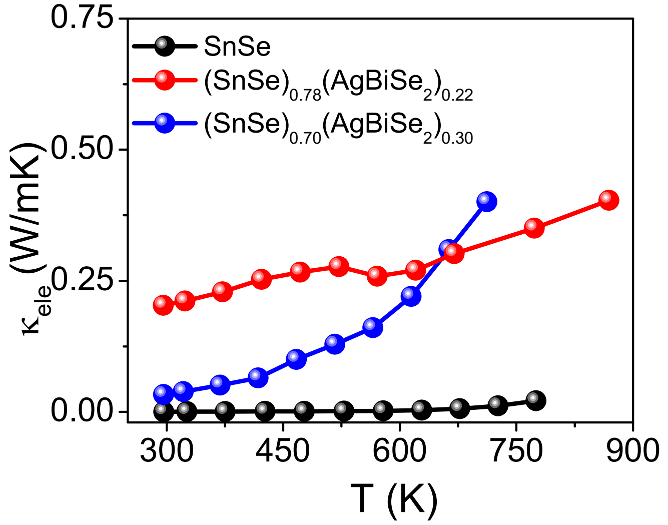

Fig. S10. Temperature dependent electrical thermal conductivity (κ_{ele}) of ball-milled polycrystalline $(SnSe)_{1-x}(AgBiSe_2)_x$ (where, x = 0, 0.22 are orthorhombic and x = 0.30 is cubic in nature) measured along parallel to the SPS pressing direction.

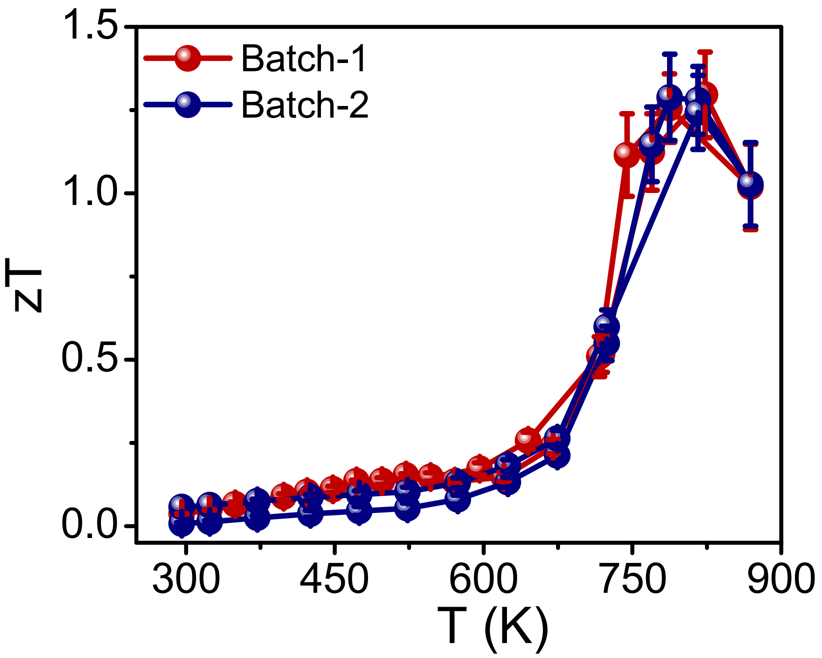

Fig. S11. The reversibility and reproducibility of the thermoelectric figure of merit of the SPS processed ball milled orthorhombic $(SnSe)_{0.78}(AgBiSe_2)_{0.22}$ sample measured for different batches (synthesized separately) with the heating cooling cycles. The zT is measured along the parallel to SPS pressing direction.

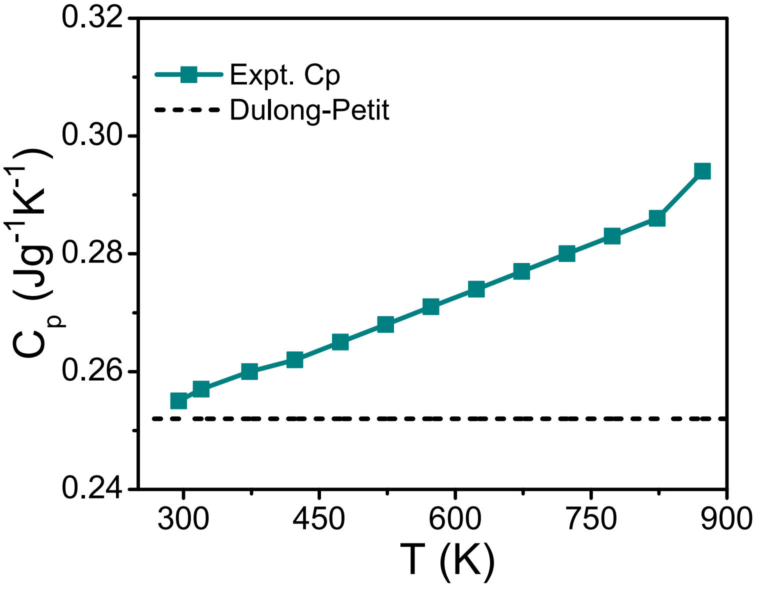

Fig. S12. Typical heat capacity (C_p) of polycrystalline $(SnSe)_{1-x}(AgBiSe_2)_x$ samples along with the Dulong-Petit C_p value of SnSe.

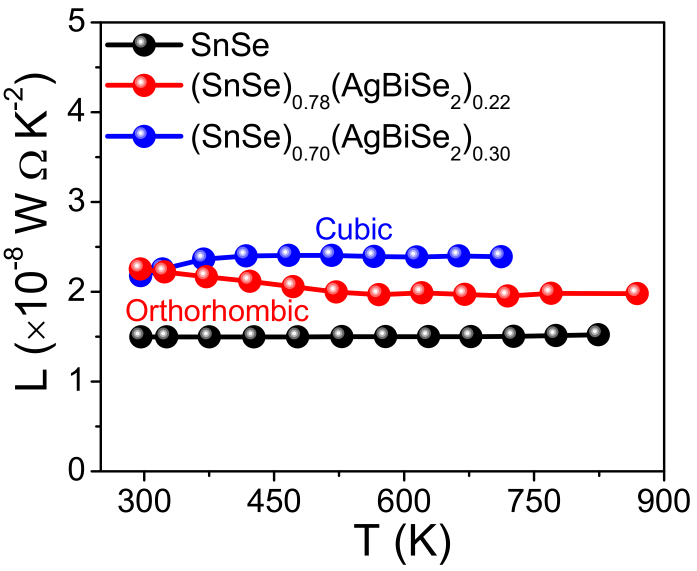

Fig. S13. Temperature dependent Lorenz number of ball-milled polycrystalline $(SnSe)_{1-x}(AgBiSe_2)_x$ (where, x = 0, 0.22 are orthorhombic and x = 0.30 is cubic in nature) along parallel to the SPS pressing direction.

Table S1. Structural parameters of Rietveld refinement for orthorhombic $(SnSe)_{0.78}(AgBiSe_2)_{0.22}$ sample.

Space group: *Pnma*; a = 11.50 Å, b = 4.15 Å, c = 4.44 Å, α = β = γ= 90°

Constituent Elements	x/a	y/b	z/c	U_{iso} (Å^2)	Occupancy	χ^2
Sn	0.8564(5)	0.25	0.4755(4)	0.1148(4)	0.63(2)	2.37
Ag	0.8564(5)	0.25	0.4755(4)	0.1148(4)	0.21(2)	
Bi	0.8564(5)	0.25	0.4755(4)	0.1148(4)	0.16(2)	
Se	0.1181(4)	0.25	0.1091(5)	0.0380(5)	1	

R-factors: R_{wp}: 12.61; R_{exp}: 8.19

Table S2. Structural parameters of Rietveld refinement for cubic $(SnSe)_{0.70}(AgBiSe_2)_{0.30}$ sample.

Space group: $Fm\bar{3}m$; a = b = c = 5.8819 Å, α = β = γ= 90°

Constituent Elements	x/a	y/b	z/c	U_{iso} (Å^2)	Occupancy	χ^2
Sn	0.0	0.0	0.0	0.1558(3)	0.54(2)	4.43
Ag	0.0	0.0	0.0	0.1558(3)	0.27(2)	
Bi	0.0	0.0	0.0	0.1558(3)	0.19(2)	
Se	0.5	0.5	0.5	0.0815(4)	1	

R-factors: R_{wp}: 11.93; R_{exp}: 5.6

Table S3. Charge carrier concentration and mobility of the ball milled and SPS processed polycrystalline $(SnSe)_{1-x}(AgBiSe_2)_x$ (x = 0, 0.22, 0.30) samples.

Composition	Carrier concentration n (cm^{-3})	Carrier Mobility μ (cm^{2}V^{-1}s^{-1})
SnSe	8.9×10^{17} (*p*-type)	8.3
$(SnSe)_{0.78}(AgBiSe_2)_{0.22}$ (Orthorhombic)	8.2×10^{19} (*p*-type)	20.8
$(SnSe)_{0.70}(AgBiSe_2)_{0.30}$ (Cubic)	6.17×10^{18} (*n*-type)	54.6

Table S4. Densities of the ball milled and SPS processed polycrystalline $(SnSe)_{1-x}(AgBiSe_2)_x$ (x = 0, 0.22, 0.30) samples.

Composition	Density (gm cm^{-3})
SnSe	5.99
$(SnSe)_{0.78}(AgBiSe_2)_{0.22}$ (Orthorhombic)	6.01
$(SnSe)_{0.70}(AgBiSe_2)_{0.30}$ (Cubic)	5.93

Table S5. Comparison of κ_{lat} of various high performance SnSe based polycrystals.

Product	κ_{lat} (W m^{-1} K^{-1})	T (K)	Reference
**$(SnSe)_{0.78}(AgBiSe_2)_{0.22}$**	**0.19**	**773**	**(This Work)**
$AgSnSbSe_{1.5}Te_{1.5}$	0.32	723	12
$Sn_{0.50}(AgBi)_{0.25}Se_{0.50}Te_{0.50}$	0.24	820	13
$(Na_{0.01}Sn_{0.99}Se)$-5%(PbSe)	0.11	773	14
$Sn_{0.40}(AgBi)_{0.30}Se$	0.45	842	15
$K_{0.01}Sn_{0.99}Se$	0.20	773	16
$Ag_{0.01}Sn_{0.99}Se_{0.85}Se_{0.15}$	0.11	825	17
$Sn_{0.95}Se$	0.23	873	18
SnSe + 1mol% PbSe	0.15	873	19

REFERENCES

1 A. Banik, U. S. Shenoy, S. Saha, U. V Waghmare and K. Biswas, *J. Am. Chem. Soc.*, 2016, **138**, 13068–13075.

2 D. Sarkar, T. Ghosh, A. Banik, S. Roychowdhury, D. Sanyal and K. Biswas, *Angew. Chem. Int. Ed.*, 2020, **59**, 11115–11122.

3 S. Perumal, S. Roychowdhury, D. S. Negi, R. Datta and K. Biswas, *Chem. Mater.*, 2015, **27**, 7171–7178.

4 G. Kresse and D. Joubert, *Phys. Rev. B*, 1999, **59**, 1758–1775.

5 X. Hua, X. Chen and W. A. Goddard, *Phys. Rev. B*, 1997, **55**, 16103–16109.

6 J. P. Perdew, K. Burke and M. Ernzerhof, *Phys. Rev. Lett.*, 1996, **77**, 3865–3868.

7 A. Dal Corso, *Comput. Mater. Sci.*, 2014, **95**, 337–350.

8 A. van de Walle, P. Tiwary, M. de Jong, D. L. Olmsted, M. Asta, A. Dick, D. Shin, Y. Wang, L.-Q. Chen and Z.-K. Liu, *Calphad*, 2013, **42**, 13–18.

9 D. Gresch, G. Autès, O. V. Yazyev, M. Troyer, D. Vanderbilt, B. A. Bernevig and A. A. Soluyanov, *Phys. Rev. B*, 2017, **95**, 075146.

10 A. A. Soluyanov and D. Vanderbilt, *Phys. Rev. B*, 2011, **83**, 235401.

11 J. Bhattacharjee and U. V. Waghmare, *Phys. Rev. B*, 2005, **71**, 045106.

12 Y. Luo, S. Hao, S. Cai, T. J. Slade, Z. Z. Luo, V. P. Dravid, C. Wolverton, Q. Yan and M. G. Kanatzidis, *J. Am. Chem. Soc.*, 2020, **142**, 15187–15198.

13 H. Wang, H. Hu, N. Man, C. Xiong, Y. Xiao, X. Tan, G. Liu and J. Jiang, *Mater. Today Phys.*, 2021, **16**, 100298.

14 Y. K. Lee, Z. Luo, S. P. Cho, M. G. Kanatzidis and I. Chung, *Joule*, 2019, **3**, 719–731.

15 H. X. Wang, L. S. Mao, X. J. Tan, G. Q. Liu, J. T. Xu, H. Z. Shao, H. Y. Hu and J. Jiang, *Nano Energy*, 2018, **51**, 649–655.

16 Y. X. Chen, Z. H. Ge, M. Yin, D. Feng, X. Q. Huang, W. Zhao and J. He, *Adv. Funct. Mater.*, 2016, **26**, 6836–6845.

17 C. C. Lin, R. Lydia, J. H. Yun, H. S. Lee and J. S. Rhyee, *Chem. Mater.*, 2017, **29**, 5344–5352.

18 W. Wei, C. Chang, T. Yang, J. Liu, H. Tang, J. Zhang, Y. Li, F. Xu, Z. Zhang, J. F. Li and G. Tang, *J. Am. Chem. Soc.*, 2018, **140**, 499–505.

19 G. Tang, W. Wei, J. Zhang, Y. Li, X. Wang, G. Xu, C. Chang, Z. Wang, Y. Du and L. D. Zhao, *J. Am. Chem. Soc.*, 2016, **138**, 13647–13654.
